# Characterization of a Cytopathogenic Reporter CSFV

**DOI:** 10.3390/v13071209

**Published:** 2021-06-23

**Authors:** Carina Maria Reuscher, Lisa Schmidt, Anette Netsch, Benjamin Lamp

**Affiliations:** Institute of Virology, Faculty of Veterinary Medicine, Justus-Liebig-University, Biomedical Research Center, Schubertstrasse 81, 35392 Giessen, Germany; Carina.M.Reuscher@vetmed.uni-giessen.de (C.M.R.); Lisa.M.Schmidt@bio.uni-giessen.de (L.S.); Anette.Netsch@vetmed.uni-giessen.de (A.N.)

**Keywords:** pestiviruses, classical swine fever virus, serum virus neutralization assay, cytopathogenicity, reporter virus, fluorescence-verified plaque reduction assay

## Abstract

Cytopathogenic (cp) pestiviruses frequently emerge in cattle that are persistently infected with the bovine viral diarrhea virus (BVDV) as a consequence of RNA recombination and mutation. They induce apoptosis in infected tissue cultures, are highly attenuated in the immunocompetent host, and unable to establish persistent infections after diaplacental infections. Cp strains of BVDV have been used as naturally attenuated live vaccines and for species-specific plaque reduction tests for the indirect serological detection of BVDV. Here, we present a genetically engineered cp strain of the classical swine fever virus (CSFV). Cytopathogenicity of the strain was induced by the insertion of ubiquitin embedded in a large NS3 to NS4B duplication. The CSFV RNA genome was stabilized by the inactivation of the NS2 autoprotease, hindering the deletion of the insertion and the reversion to a wild-type genome. Additional insertion of a mCherry gene at the 5′-end of the E2 gene allowed fluorescence-verified plaque reduction assays for CSFV, thus providing a novel, cost-efficient diagnostic tool. This genetically stabilized cp CSFV strain could be further used as a basis for potential new modified live vaccines. Taken together, we applied reverse genetics to rationally fixate a typical cp NS3 duplication in a CSFV genome.

## 1. Introduction

The classical swine fever virus (CSFV) is the causative agent of classical swine fever (CSF), formerly termed hog cholera. CSFV has been eradicated in most developed countries, but despite longstanding eradication efforts by the OIE (World Organization for Animal Health), CSF is still an economically important disease. Recent outbreaks of CSFV in the domestic pig population and/or endemic situations in known wild boar reservoirs are reported from Africa, Asia, the Caribbean, Central and South America and Europe [1,2,3]. The clinical signs of CSFV infections are variable and might include fever, conjunctivitis, respiratory signs, constipation, diarrhea, skin hemorrhages, lethargy, neurological symptoms, abortion, stillbirth and death [3]. Depending on the virulence of the respective CSFV strain on one hand and the age and immune status of the host, on the other hand, acute, chronic or persistent forms of the disease occur, which require molecular diagnostics of the pathogen [4]. Very effective vaccines against CSF, suitable protocols for direct detection of the CSFV genome by (q)RT-PCR, and different ELISA formats for detection of CSFV-specific antibodies exist. However, since CSFV outbreaks occur mainly in less developed regions of the world, there is still a need for cost-effective diagnostic methods for the determination and quantification of neutralizing antibodies. In this study, we report on a dual reporter CSFV that enables low-cost fluorescence-verified serum virus neutralization assays (SVNA).

The CSFV is a member of the genus *Pestivirus* within the *Flaviviridae* family [5]. The small enveloped pestiviral particle contains a single-stranded, positive-sense RNA genome of about 12.3 kb [6]. This genome encodes a single open reading frame (ORF) flanked by a 5’-UTR containing an internal ribosomal entry site (IRES), and a short 3’-UTR necessary for genome replication [7]. The IRES mediates translation of a hypothetical polyprotein, which is co- and post-translationally processed by cellular and viral proteases. The structural proteins (Core, E^rns^, E1 and E2) are located in the N-terminal third of the polyprotein downstream of an autoprotease (N^pro^). The structural proteins are processed by host cell proteases (signal peptidase and signal peptide peptidase) and are not involved in genome replication [8]. The non-structural proteins p7, NS2, NS3, NS4A, NS4B, NS5A and NS5B are organized in the C-terminal region of the polyprotein and mainly processed by the viral major protease NS3. Replicative subgenomes encoding solely NS3, NS4A, NS4B, NS5A and NS5B have been described, demonstrating that expression of these non-structural proteins is sufficient to replicate the RNA genome. The uncleaved NS2-3 precursor, together with NS4A, is required for pestiviral morphogenesis [9], while mature NS3 is an integral part of the replication complex [4,10,11]. The NS3 plays a central role in the pestiviral life cycle with its N-terminal serine protease domain responsible for polyprotein processing and a C-terminal NTPase/helicase domain catalyzing NTP-dependent RNA strand separation [12]. NS3 maturation and release from its precursor protein NS2-3 is essential for viral replication and is mediated by the NS2-autoprotease. In pestiviruses, this process is regulated by the cellular protein DNAJC14, termed JIV (J-domain protein interacting with viral protein), which acts as an essential cofactor of the NS2 autoprotease.

The regulation of NS2-3 cleavage hallmarks the non-cytopathogenic (ncp) biotype of pestiviruses, which is able to establish persistent infections after diaplacental infections and does not cause cytopathic effects in cultured cells. In contrast, efficient maturation of NS3 and high-level replication are observed in the cytopathogenic (cp) biotype of pestiviruses that cannot establish persistent infections in the natural host, is highly attenuated, and induces apoptosis in cell cultures [13,14]. The emergence and characteristics of cp strains have been extensively studied in the bovine viral diarrhea virus (BVDV) in the past. Diaplacental infected calves can develop immunotolerance to BVDV and thus become persistently infected (PI) [15]. Cp variants of BVDV emerge spontaneously in these PI calves over time as a consequence of RNA recombination and/or mutation events. The cp strains, which differ only slightly in their sequence, are included in the immunotolerance and are responsible for the onset of a fatal clinical picture, termed mucosal disease, in PI calves. [16,17,18]. The molecular basis of pestiviral cytopathogenicity has been studied using isolated ncp/cp BVDV pairs. While larger amounts of mature NS3 were observed in all cp variants, minute quantities of mature NS3 are detectable in ncp BVDV solely at early timepoints after infection. Mature NS3 accelerates genome replication and thus causes elevated viral protein levels in the infected cells leading to apoptosis [19,20,21]. In these variant BVDVs, the free NS3 can either be released more efficiently from the NS2-3 precursor via an increase in NS2 autoprotease activity or be generated independently of the NS2 autoprotease by additional processing signals. Increased NS2 autoprotease activity can be caused by mutations within the NS2 protease domain (BVDV strain CP7) [22] or genomic integration and expression of the cellular mRNA of the essential cofactor JIV (BVDV strain NADL) [16]. The insertion of a ubiquitin sequence between NS2 and NS3 (BVDV strain Osloss) [23], a duplication of the NS3 gene or deletion of NS2 resulting in defective interfering (DI) subgenomes have also been documented [24,25,26,27]. An authentic, functional N-terminus of NS3 can be generated by cofactor independent autoproteases, such as N^pro^ (BVDV strain cpPE515), or by the insertion of other cellular processing signals, such as ubiquitin (BVDV strain CP1) [28]. Cp BVDV strains have been commonly used as naturally attenuated virus variants in commercial live vaccines to control and prevent BVDV infections. The inoculation of an immunocompetent host with a cp BVDV produces stable, long-lasting immune protection due to high antigen expression [29,30]. 

In contrast to BVDV, helpervirus-independent cp genomes of CSFV have not been found in the field [31,32]. Thus far, all cp isolates obtained from CSFV infected pigs represented defective interfering particles, which were trans-complemented by an ncp CSFV helper strain [7,33,34,35]. However, helpervirus-independent cp strains of CSFV have already been generated in vitro using RNA recombination and/or reverse genetics systems. A first helpervirus-independent cp CSFV strain (CP G1) was generated by non-homologous RNA recombination in cells infected with ncp CSFV and transfected with replication-deficient DI-like subgenomes. This elegant strategy produced cp CSFVs with a duplicated viral non-structural protein cassette inserted behind a ubiquitin coding sequence at the C-terminus of the polyprotein (BVDV CP14-like). Sadly, these viruses were genetically unstable and split up into different DI subgenomes [36]. In another approach, a reverse genetics system for CSFV Alfort/Tuebingen was employed to generate a cp CSFV, which expresses the JIV gene (CSFV Alfort-JIV). This cp virus contained a complex genomic insertion derived from the cp BVDV strain CP8. CSFV Alfort-JIV was genetically stable and characterized in animal experiments as a suitable vaccine candidate that is highly attenuated in the natural host [37]. 

Reporter CSFVs were used decades ago for the direct detection of viral replication without the use of immunolabeling (reviewed in [38]). Studying the biology of non-cytopathogenic viruses, screening antiviral drugs and studying viral receptors have been facilitated by genetically engineered CSFV reporter strains. Beginning with Moser and colleagues who generated CSFVs expressing chloramphenicol acetyltransferase (CAT) in the late 1990s, different tags have been studied [39]. Reporter CSFVs were genetically engineered to be carrying a luciferase gene [40], the 11-amino-acid subunit gene derived from the NanoLuc luciferase assay [41], the enhanced green fluorescent protein gene [42], or a tetracysteine-tag for biarsenically labeling [43]. In a complementary approach, a reporter cell line has been generated that stably expresses the enhanced green fluorescent protein (eGFP) fused in-frame to a quenching peptide via a recognition sequence of the CSFV NS3 protease. This quenching peptide was specifically cleaved by the NS3 protease during CSFV infection, allowing indirect monitoring of NS3 expression by a dark to green fluorescent phenotype of the cells [44]. Most reporter genes were inserted at the 5′-end of the N^pro^ gene. Since the N^pro^ is an intrinsic unstable protein [45], even short-term association during polyprotein processing lowers the expression levels. Reporter pestiviruses with tagged envelope proteins were first presented in 2019 [46,47]. E2 fusion protein reporter viruses showed stronger reporter activity because the E2 is stable and accumulates in infected cells. Such an E2 reporter pestivirus was recently used in serological assays to determine LINDA pestivirus seroprevalence [48]. Considering that cp pestiviruses have much stronger protein expression than ncp pestiviruses, we hypothesized that reporter activity for E2 reporter genes would be further increased in the context of cp viruses. Here, we present a stable reverse genetics BAC-plasmid system for a cp CSFV encoding a ubiquitin insertion embedded in an NS3 to NS4B duplication. After the additional insertion of the foreign fluorescent protein gene mCherry, the cp CSFV allowed fluorescence-verified plaque reduction assays. Apart from providing a new cost-effective diagnostic tool, the cp CSFV can also be used in basic research, vaccine testing and development of new live attenuated vector vaccines. 

## 2. Materials and Methods

### 2.1. Cells and Viruses

SK-6 cells [49] kindly provided by M. Schweizer (Institute of Virology and Immunoprophylaxis, Mittelhäusern, Switzerland), were grown in Dulbecco’s modified Eagle’s medium (DMEM, Biowest, Nuaillé, France) supplemented with 10% heat-inactivated fetal calf serum (FCS, Corning, Tewksbury, MA, USA; negatively tested for pestiviruses), and 100 U/mL penicillin and 100 µg/mL streptomycin. Cells were maintained at 37 °C and a CO_2_ concentration of 5%. Mutagenesis of CSFV was performed modifying an established reverse genetics system for the CSFV strain Alfort/Tuebingen [50]. The nucleotide and amino acid numbers of CSFV throughout this study refer to the sequence of this parental ncp CSFV strain (GenBank J04358.2). Infections of SK-6 cells were performed in serum-free DMEM with the indicated multiplicity of infection (MOI) for one hour before a fresh medium including FCS was added. Plaque and foci size were determined after the infection of SK-6 cells with 10 focus/plaque-forming units (FFU/PFU) per well for two hours. To inhibit the spread of the viruses through the medium in plaque assays, the supernatant was removed and replaced by fresh DMEM containing FCS and 1% carboxymethyl cellulose. The infected cells of 50 individual foci were counted, the mean focus size was calculated, and the 95% confidence interval was determined to analyze the virus spread of cp CSFV in cultured cells.

### 2.2. Generation of CSFV cDNA Clones

A cellular ubiquitin insertion cassette was amplified from the cp BVDV strain CP Rit encoding an N-terminally truncated (3 aa) ubiquitin moiety together with parts of the ribosomal protein S27a [51]. CP Rit is a temperature-sensitive cp BVDV strain that is widely used for vaccination (Rispoval^®^ D-BVD, CP 4350; Pfizer Animal Health, New York, NY, USA). The cellular insertion was amplified using the oligonucleotides Ubi*_forw (5′-GAGAATGGCAAAATCAGTCGCCTTC-3′) and Ubi*_rev (5′-CCCACCACGAAGTCTCAACACTAG-3′) and the One-Step OneTaq RT-PCR Kit (NEB, Ipswich, MA, USA). After sub-cloning in a T-vector (pGem-T, Promega Corporation, Madison, WI, USA), the gene was amplified by extension PCR with suitable nucleotide overhangs for an assembly reaction with oligonucleotides CSFV-353-Ubi*-GA_forw (5′-ATCTCTGCTGTACATGGCACATGGAGGTACATGGCACATGGGAGAATG-3′ and CSFV-5163-Ubi*-GA_rev (5′-CTTGCAAACAGCTGGCCCCCCACCACGAAGTCTCAAC-3′). The CSFV cDNA clone was used for the generation of a replication-competent DI9-like subgenome. Therefore, the pBR322 plasmid backbone with the 5′-UTR and the non-structural protein region of CSFV was amplified using a Phusion reaction together with the oligonucleotides CSFV-378_rev (5′-CTCCATGTGCCATGTACAGCAGAGAT-3′) and CSFV-5140_forw (5′-GGGCCAGCTGTTTGCAAGAAGG-3′). This in vitro plasmid assembly reaction (NEBuilder, NEB) generated the plasmid pR4 (cp CSFV-DI-Ubi*).

The cp CSFV strain containing a duplication of the non-structural proteins NS3 to NS4B downstream of the cellular insertion cassette was constructed in a bacterial artificial chromosome (BAC) backbone using a three-piece assembly approach. Therefore, a BAC-backbone (pBeloBAC11, NEB) with a SP6 promoter was amplified by a Phusion polymerase reaction using the oligonucleotides BAC-SP6_rev (5′-GTATAGTGTCACCTAAATCGTTACAATTCACTGGCCGTCG-3′) and CSFV-12271-BAC-GA_forw (5′-GACTAAGGTAATTTCCTAACGGCCCTAAATAGCTTGGCGTAATCATGGTC-3′). Then, the CSFV cDNA was amplified with an SP6 promoter from the 5′-end to the desired insertion locus within the N-terminal part of the NS4B gene using the plasmid p447 as a template with the oligonucleotides SP6_forw (5′-ACGATTTAGGTGACACTATAG-3′) and Ubi*-CSFV-7776-GA_rev (5′-GAAGGCGACTGATTTTGCCATTCTCTTGTTGTGTTTCTGTGTCTCCTG-3′). The plasmid pR4 (cp CSFV-DI-Ubi*) was used as a template to amplify the ubiquitin insertion and the upstream non-structural proteins NS3 to NS5b together with the 3′-UTR with the help of the oligonucleotides Ubi*_forw and CSFV-12295_rev (5′-GGGCCGTTAGGAAATTACCTTAGTC-3′). The DNA fragments were assembled, producing the cDNA plasmid pR6 (cp CSFV-Ubi*). The pBeloBAC11 derived plasmid pR6 was transformed in *E. coli*, strain ER2420 (NEB), which has an EcoK r m, McrBC, Mrr Dam and Dcm background. The plasmid pBeloBAC11 carries a gene encoding resistance to chloramphenicol (Cam). After transformation and to retain the plasmid, cells were grown with 17 µg/mL Cam. 

The cp CSFV-Ubi* was stabilized by mutagenesis, causing the amino acid exchange C_1512_A in the NS2 protein that prevents NS2-3 cleavage by the inactivation of the NS2 autoprotease. Mutagenesis was performed using PCR and DNA assembly. The oligonucleotides CSFV-4891-A1512_forw (5′-GGACCACCAGTGGTCGCCGGTATGACCCTAGCCGATTTC-3′) and CSFV_12295_rev were used for the amplification of one fragment, while CSFV-4932-A1512_rev and CSFV-12271-BAC-GA_forw were used for the other. The resulting plasmid was named pR14 (cp CSFV-Ubi*-C_1512_A). 

**Figure 1:**
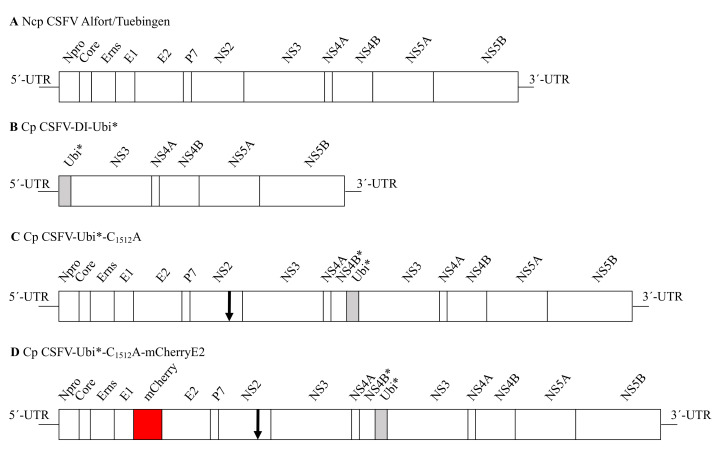
Scheme of the genomic organization of the infectious CSFV cDNA clones. (**A**) Ncp CSFV Alfort/Tuebingen with a typical pestiviral genome organization. (**B**) Cp CSFV-DI-Ubi* with a deletion of the 5’-end of the ORF (N^pro^ to NS2) replaced by a cellular insertion cassette encoding a truncated ubiquitin (Ubi*, marked in gray), which mediates the generation of the authentic NS3 N-terminus. (**C**) Cp CSFV-Ubi*-C_1512_A with duplication of NS3 to NS4B* genes and an insertion of Ubi*. The arrow indicates a stabilizing mutation C_1512_A that inactivates the NS2 autoprotease. (**D**) Cp CSFV-Ubi*-C_1512_A-mCherryE2 as a dual reporter construct with an insertion of the fluorescent protein mCherry (highlighted in red) at the 5’-end of the E2 gene, a duplication of NS3 to NS4B* genes, the insertion of Ubi*, and the stabilizing mutation C_1512_A. Stars indicate truncated proteins.

For the insertion of the mCherry gene at the 5′-end of the E2 gene of CSFV, a three-piece assembly reaction was designed. The complete BAC-backbone, the 5´-UTR and the coding region of N^pro^, E^rns^ and E1 were amplified from pR14 using the oligonucleotides CSFV-12271-BAC-GA_forw and CSFV-2442_rev (5′-CCGCCCTTGTGCCCCGGTCACCAGCAGCAGCC-3′). The E2 gene and downstream CSFV sequences, including the cellular insertion cassette and duplicated non-structural proteins, were amplified using the oligonucleotides CSFV-2443_forw (5′-CTAGCCTGTAAGGAAGACTACAGGTATGCGATC-3′) and CSFV-12295_rev. A mCherry coding DNA fragment was amplified using extension PCR to provide complementary sequences using the oligonucleotides CSFV-2424-mCherry-GA_forw (5′-GACCGGGGCACAAGGGCGGGTGAGCAAGGGCGAGGAGGATAAC-3′) and CSFV-2465-mCherry-GA_rev (5′-CTGTAGTCTTCCTTACAGGCTAGCTTGTACAGCTCGTCCATGCCG-3′). The PCR products were purified and assembled using a NEBuilder reaction. The resulting plasmid was termed pR31 (cp CSFV-Ubi*-C_1512_A-mCherryE2). Mutagenesis of the constructs was validated using a commercial Sanger sequencing service (Microsynth, Balgach, Switzerland). A scheme of the respective constructs is shown in Figure 1. A table of all oligonucleotides used in this study is presented in Table 1.

### 2.3. Virus Rescue

PCR products were used as the template for SP6-dependent in vitro RNA synthesis. The DNA templates were amplified from the respective plasmids using Phusion polymerase (NEB) together with the oligonucleotides SP6_forw and CSFV-12295_rev. A total of 250 ng of phenol-chloroform purified PCR products were used as the template for in vitro transcription reactions as previously described [12]. After synthesis, 50 µL of the SP6 transcription reactions was DNase digested. The RNA was purified with the RNeasy Mini Kit (QIAGEN, Hilden, Germany), eluted in RNase-free water, and diluted in water to a final concentration of 0.25 µg/µL. SK-6 cells were transfected with 2.5 µg of the synthetic RNA by electroporation with 150 kV and 950 µF in a 0.2 mm cuvette (GenePulser, Bio-Rad, Feldkirchen, Germany), and incubated for the indicated time until viral progeny and/or cells were harvested. The Phusion polymerase is one of the most accurate thermostable polymerases available, with an error rate of about 4.4 × 10^7^ to 9.5 × 10^7^, according to the manufacturer’s product information. This error rate comes close to the range of the DNA plasmid replication in *E. coli*, which has an error rate between 1 × 10^9^ and 1 × 10^11^, depending on genotype and growth conditions [52]. However, in vitro transcription of DNA using SP6 or T7 RNA polymerases typically results in high error rates, estimated to be about 2 × 10^4^ [53]. Therefore, the virus genomes were analyzed by RT-PCR and/or consensus Sanger sequencing after rescue. These analyses included amplification of the first third of the genome up to the foreign gene insertion using the oligonucleotides CSFV-RT-qPCR-99_forw and CSFV-5694_rev, testing the stability of the Ubi* insertion using the oligonucleotides Ubi*_forw and Ubi*_rev and visualizing the mCherry foreign gene using the oligonucleotides mCherry_forw and mCherry_rev. We further re-cloned parts of the genomes of cp CSFV-Ubi*-C_1512_A and cp CSFV-Ubi*-C_1512_A-mCherryE2 to analyze the occurrence of mutations during passaging.

### 2.4. Indirect Immunofluorescence Assay and Western Blot

Indirect immunofluorescence assays were performed as previously described [54]. Briefly, the cells were fixed with 4% paraformaldehyde for 20 min at 4 °C, permeabilized with 1% (*v*/*v*) Triton-X 100 (Merck, Darmstadt, Germany) in PBS, and stained with the monoclonal antibody (Mabs) 8.12.7 anti NS3 [55] or A18, which is reactive against E2. Goat anti-mouse IgG conjugated with Cy3 (Dianova, Hamburg, Germany) or goat anti-mouse IgG conjugated with FITC (Dianova) were used as secondary antibodies. Cell nuclei were counterstained with DAPI (Thermo Fisher Scientific, Waltham, MA, USA) at a concentration of 1 µg/mL for 5 min at room temperature. For Western blotting, SK-6 cells were transfected with synth, etic RNAs to ensure complete and synchronized infection and harvested at 24 h post-transfection. Proteins were separated in 7.5% (*wt/vol*) polyacrylamide tricine gels and transferred onto nitrocellulose membranes (Pall, Pensacola, FL, USA). The membranes were blocked with 5% (*wt/vol*) skim milk (Carl Roth, Karlsruhe, Germany) in phosphate-buffered saline (PBS) with 0.05% (*v*/*v*) Tween-20 (Invitrogen, Karlsruhe, Germany). The murine Mabs A18 anti-CSFV E2, 8.12.7 anti NS3, GL5A1 anti NS5A [50] and E5D8F anti mCherry [56] (Cell Signaling Technology, Danvers, MA, USA) were used for antigen detection as indicated. Mabs were visualized with the help of peroxidase-coupled secondary antibodies against murine IgG (Dianova). ECL-Prime was applied as a Western blotting chemiluminescence reagent (GE Healthcare, Chicago, IL, USA), and photon emission was recorded with an imaging system (ChemiDoc, Bio-Rad).

### 2.5. Serum Virus Neutralization Assays (SVNA)

SVNAs were performed as described previously [57]. Serum dilutions were prepared in triplicate in DMEM without FCS in 96-well cell culture plates (STARLAB, Hamburg, Germany) by adding 25 µL of the serum of interest to 100 µL of medium provided. After thorough mixing, 25 µL of the dilution was then again taken and pipetted into the next dilution level. The ncp CSFV strain Alfort/Tuebingen and the cp CSFV-Ubi*-C_1512_A-mCherryE2 were diluted to a final titer of 150 TCID_50_/50 µL. The test virus was added to the 1:5 serum dilution series and incubated at 37 °C for 2 h. A total of 1 × 10^4^ SK-6 cells were seeded directly into the wells containing the pre-incubated serum/virus mixture and grown for 48 h. Defined positive and negative reference antisera, kindly provided by the EU and OIE Reference Laboratory for Classical Swine Fever (Institute of Virology, Department of Infectious Diseases, University of Veterinary Medicine, Hannover, Germany), serum toxicity controls (serum dilution 1/5), cell controls and virus back titration controls were included in each assay. Cells were fixed with 4% paraformaldehyde in PBS for 20 min at 4 °C after 48 h, when strong fluorescence signals and cytopathogenic effects were visible in the control wells with the cp CSFV-Ubi*-C_1512_A-mCherryE2 back titrations. All CSFV infected cells were stained with the E2-specific Mab A18, and a FITC labeled goat anti-mouse IgG for comparison. The SVNA reactions were analyzed using a fluorescence microscope (Olympus IX70 fluorescence microscope; OLYMPUS, Hamburg, Germany). The 50% neutralization dose (ND_50_/mL) was calculated for each virus-serum combination using the Spearman–Kaerber method.

### 2.6. RT-qPCR

RNA extractions were performed using a volume of 140 µL (cell-free culture supernatant or cell lysates). Total RNA was extracted using the QIAamp Viral RNA Mini Kit (QIAGEN, Hilden, Germany) or the RNeasy Mini Kit (QIAGEN) according to the manufacturer’s instructions on a QIAcube Connect device (QIAGEN). RT-qPCRs were performed on a StepOne real-time PCR system (Applied Biosystems, Waltham, MA, USA) using the Luna Universal Probe One-Step RT-qPCR Kit (NEB). CSFV specific primers and probes were used as previously described [58] together with a synthetic RNA standard for absolute quantification.

## 3. Results

### 3.1. The Cellular Insertion Cassette of BVDV CP Rit Is Active as a Processing Signal on CSFV NS3

**Figure 2:**
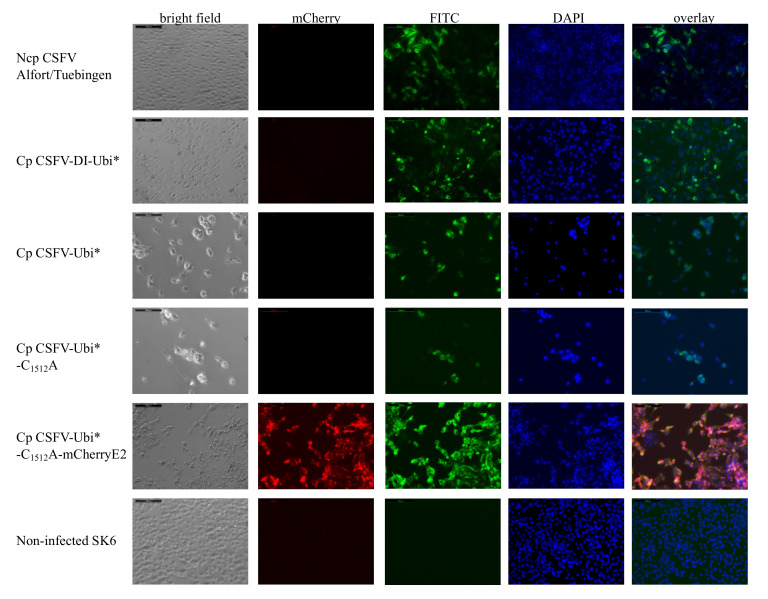
Indirect immunofluorescence assay of SK-6 cells transfected with the different ncp and cp CSFV strains. SK-6 cells were transfected with synthetic RNA of the respective virus genomes as indicated on the left. Cytopathic effects of the cp CSFVs were documented in bright-field images. The CSFV infected cells were stained with the NS3-specific Mab 8.12.7 that was visualized by a FITC-conjugate. Nuclei were counterstained with DAPI, and mCherry fluorescence was imaged. The fluorescence images were combined in overlay for direct comparison. All images were taken at 20x magnification, including the virtual scale bar (200 µm).

The cp BVDV strain CP Rit has incorporated a cellular mRNA sequence coding parts of the ribosomal protein S27a fused with an N-terminally truncated ubiquitin moiety [51]. This fusion protein mediates the NS2 independent release of mature NS3 in the BVDV strain and is the molecular basis for enhanced replication, strong protein expression, cytopathogenicity in cell culture, and attenuation in the natural host. We generated a CSFV replicon with the CP Rit insertion (Ubi*) at the 5′-end of the ORF to test the activity of this ubiquitin insertion as a processing signal for CSFV NS3. The resulting replicon, termed cp CSFV-DI-Ubi*, was constructed in analogy with the well-characterized CSFV-DI-N^pro^ that autoproteolytically processed NS3 very efficiently [50]. We observed strong cytopathic effects starting 24 h after transfection of the 7.8 kb long RNA of the cp CSFV-DI-Ubi* subgenome as shown before in CSFV-DI-N^pro^ transfected cells. An indirect immunofluorescence test using Mab 8.12.7 anti NS3 showed a strong cytoplasmic fluorescence signal in the dead cells 48 h post-transfection. The bright NS3 dependent staining within the cytoplasm together with the death of the transfected cells is shown in Figure 2, indicating a high-level protein expression of this subgenome, proving the activity of Ubi*.

### 3.2. Cp CSFV-Ubi* Is Stabilized by an Inactivation of the NS2 Protease

We generated a helper virus-independent cp CSFV strain by inserting Ubi* downstream of codon Q_2468_ (aa 132 of NS4B) of CSFV. In analogy to the organization of the BVDV CP Rit, the genes from NS3 to NS4B were duplicated and embedded in the complete non-structural protein module (NS3-NS5B) downstream of the Ubi*. The cDNA clone of cp CSFV-Ubi* with a total genome length of 15.2 kb was constructed in a BAC backbone to ensure genetic stability considering the homologous duplication of more than 2.5 kb. Template cDNA was produced by long-range PCR, and synthetic RNA was transcribed via SP6-polymerase. Already 24 h after transfection of the synthetic RNA of cp CSFV-Ubi* into SK-6 cells, the first cytopathic effects were observed, and the cellular monolayer was largely destroyed within 48 h. The high replication and protein expression levels of the cp CSFV-Ubi* were demonstrated by strong fluorescence signals in immunofluorescence staining of the NS3 antigen (Figure 2). 

**Figure 3:**
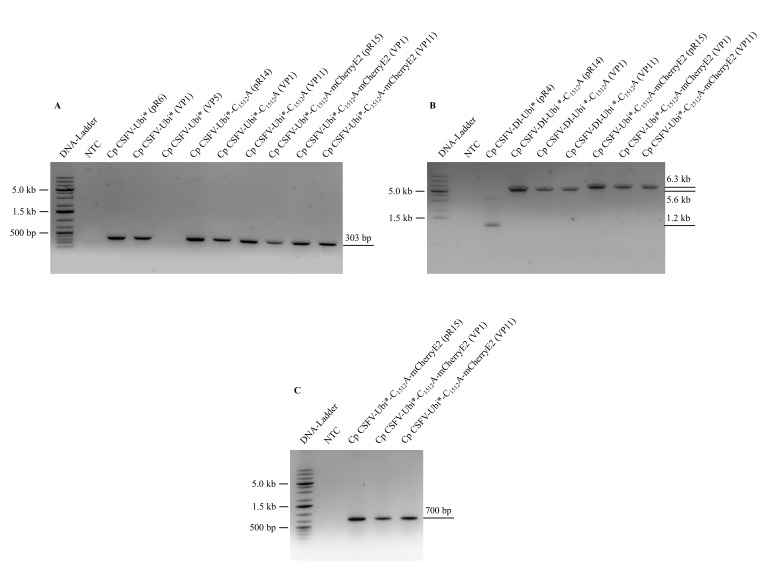
RT-PCR analysis of cp CSFV stability. (**A**) The Ubi* gene was amplified by RT-PCR from total RNA of the infected cells of the indicated virus passages (VP1, VP5 or VP11) using the oligonucleotides Ubi*_forw and Ubi*-rev. (**B**) The 5’-end of the CSFV genome was amplified using CSFV-RT-qPCR-99_forw and CSFV-5694_rev to detect the emergence of subgenomic DIs. Note the size differences of the PCR products. (**C**) The mCherry gene was amplified by RT-PCR using oligonucleotides mCherry_forw and mCherry_rev. A no-template control (NTC) and plasmid DNA positive controls (pR4, pR6, pR14 and pR15) were included for each assay. The band size of a DNA ladder (GeneRuler 1 kb Plus, Thermo Scientific) is indicated on the left side, while the RT-PCR product size is shown on the right side.

The cp CSFV-Ubi* virus was passaged on naïve SK-6 cells to analyze the stability of the genetically engineered virus. While complete lysis of the cellular monolayer was observed within the first four passages, surviving cells remained attached on the cell culture surface in virus passage five. These cells were harvested by trypsinization, seeded again, and used for the characterization of the CSFV revertant. Immunofluorescence staining with the Mab A18 against E2 verified the infection with an ncp CSFV, which presumably evolved from cp CSFV-Ubi* by homologous recombination and protected the cells from cp virus infection by superinfection exclusion. RT-PCR analysis demonstrated the loss of the Ubi* gene shown in Figure 3A, lane 5. Sanger sequencing of the NS4B gene revealed a wild-type-like ncp CSFV genome that had precisely deleted our Ubi* insertion and NS gene duplication. The wild-type-like NS4B sequence obtained from the passage 5 infected cells is presented in Supplementary Figure S1.

Recognizing this apparent instability of our construct, we searched for a way to hinder homologous recombination or the emergence of wild-type-like CSFV phenotypes following deletions of the insertion. Because even complete codon exchange in the duplicated region does not necessarily stabilize such a virus, we decided to exchange the active cysteine residue (aa 1512) within the NS2 protease domain against an alanine. This mutation inactivates the NS2 autoprotease and thus prevents NS2-3 cleavage so that C_1512_A mutants are not able to replicate [59]. Accordingly, homologous recombination and deletion of the Ubi* insertion could still happen but would generate replication-incompetent ncp CSFV-C_1512_A genomes. We introduced the mutation into the genome and, as expected, observed that CSFV-Ubi*-C_1512_A was stably inducing complete cell lysis even after eleven virus passages on SK-6 cells. Therefore, the virus supernatant from passage 11 was tested in dilution series and the infected cells were stained with Mab 8.12.7 anti NS3. RT-PCR analyses with Ubi*-forw and Ubi*_rev are shown in Figure 3A, demonstrating that the Ubi* insertion in cp CSFV-Ubi*-C_1512_A was retained in viral passage 11 (lane 7). Furthermore, RT-PCR analyses with CSFV-RT-qPCR-99_forw and CSFV-5694_rev revealed no evidence for co-passage of subgenomic DIs, as no 5’-end truncated fragment can be observed in Figure 3B, lane 6.

### 3.3. Cp CSFV-Ubi*-C_1512_A-mCherryE2 as a Stable Double Reporter Virus

To generate a new and cost-efficient diagnostic tool for indirect CSFV detection, we constructed a dual reporter virus suitable for fluorescence-verified plaque reduction assays. Therefore, the helpervirus-independent stable cp CSFV-Ubi*-C_1512_A strain was genetically labeled using the insertion of a mCherry gene at the 5′-end of the E2 gene as presented earlier [46]. The virus cp CSFV-Ubi*-C_1512_A-mCherryE2 was rescued from synthetic RNA by electroporation and analyzed in virus passages. The mCherry-E2 fusion protein in cp CSFV-Ubi*-C_1512_A-mCherryE2 is fully capable of virion morphogenesis, receptor binding, and host cell infection as demonstrated by its intact infection cycle. As in the cp CSFV-Ubi*, the first cytopathic effects were observed 24 h post-transfection, but viral spread in the infected culture was notably slower in direct comparison. A strong fluorescence of mCherry was observed 24 to 48 h after transfection, and the cellular monolayer was largely destroyed within 48 h post-transfection, as shown in Figure 2. 

To verify that the observed fluorescence signals arise due to the mCherry-E2 expression, indirect immunofluorescence using Mab 8.12.7 anti-CSFV NS3 and FITC conjugated goat anti-mouse IgG was applied. By overlaying the signals, a close correlation between red and green fluorescence was documented. However, it must be mentioned that signal amplification in immunohistochemical staining results in a very high sensitivity. Therefore, some cells at the margin of the plaques of cp CSFV-Ubi*-C_1512_A-mCherryE2, which show no or only very weak mCherry fluorescence signals, can be stained with CSFV specific antibodies. 

As the parental cp, CSFV-Ubi*-C_1512_A, the cp CSFV-Ubi*-C_1512_A-mCherryE2 was stable on SK-6 cells for 11 passages. The RT-PCR analyses of cp CSFV-Ubi*-C_1512_A-mCherryE2 presented in Figure 3A showed the preservation of the Ubi* insertion in virus passage 11 (lane 11). The stability of the mCherry insertion can be inferred from the size of the genomes 5’-end in the RT-PCR amplicon of the first third of the genome (Figure 3B, lane 9). Furthermore, direct RT-PCR amplification of the mCherry gene is shown in Figure 3C (lane 5) using the oligonucleotides mCherry_forw and mCherry_rev to specifically analyze the stability of the foreign gene insertion in the structural protein region. 

### 3.4. Characterization of Viral Replication

**Figure 4:**
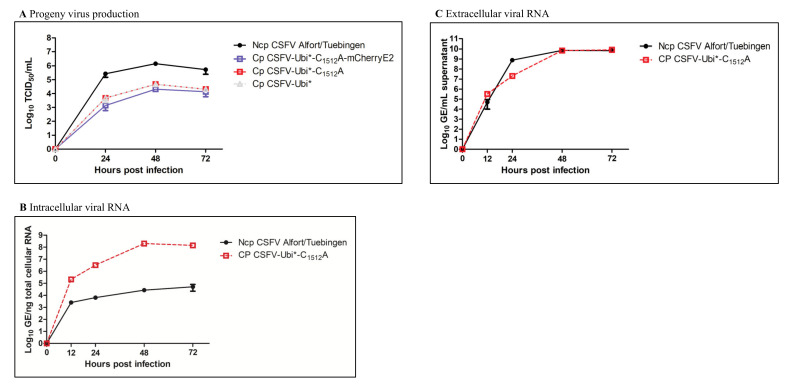
Characterization of cp CSFV replication. A monolayer of SK-6 cells was infected with an MOI of 0.01. One hour after infection, the cells were washed twice, and a fresh cell culture medium was given. (**A**) Cell culture growth of ncp CSFV Alfort/Tuebingen, cp CSFV-Ubi*, cp CSFV-Ubi*-C1512A and cp CSFV-Ubi*-C1512A-mCherryE2. Cell culture supernatant samples were taken after 24, 48 and 72 h to analyze the progeny virus production and titrated on naïve SK-6 cells. Each titration was performed in triplicates, and TCID50/mL was calculated using the Spearman–Kaerber algorithm. No infectious virus was found at the timepoint 0 h (limit of detection >1.6 × 10^2^ TCID50/mL. (**B**) Intracellular viral RNA levels. Viral RNA genome equivalents were measured per ng total cellular RNA 0, 12, 24, 48 and 72 h post infection in biological replicates. The qRT-PCR reactions were performed in triplicates. (**C**) Extracellular viral RNA levels. Viral RNA genome equivalents were measured 0, 12, 24, 48 and 72 h post infection and projected to 1 mL of cell culture supernatant. The qRT-PCR reactions were performed in triplicates. Error bars represent positive and negative standard deviations.

Progeny virus production and RNA replication were analyzed to characterize the cp CSFV strains in more detail. Naïve SK-6 cells were infected with the respective viral strains using a multiplicity of infection (MOI) of 0.01 to study the viral spread and to generate viral growth curves. Progeny virus titers were measured 24, 48 and 72 h after inoculation, as presented in Figure 4A. Cp CSFV-Ubi*, cp CSFV-Ubi*-C_1512_A, and cp CSFV-Ubi*-C_1512_A-mCherryE2 showed reduced progeny virus titers in direct comparison to the wild-type ncp CSFV Alfort/Tuebingen. In the experiment, ncp CSFV produced a peak titer of 1 × 10^6^ TCID_50_/_mL_ 48 h post infection, while cp CSFV-Ubi*, cp CSFV-Ubi*-C_1512_A and cp CSFV-Ubi*-C_1512_A-mCherryE2 released about 2 log_10_ lower titers of progeny virus in the cell culture supernatant. No effect of the C_1512_A mutation on the progeny virus production was observed comparing cp CSFV-Ubi* and cp CSFV-Ubi*-C_1512_A. The insertion of the mCherry gene additionally reduced infectious virus production by a factor of two in comparison to cp CSFV-Ubi*. We also measured intra- and extracellular viral RNA levels in ncp CSFV Alfort/Tuebingen and cp CSFV-Ubi*-C_1512_A 12, 24, 48 and 72 h post infection (Figure 4B,C). Viral RNA levels within the infected cells were about 1000 to 10,000-times higher in cp CSFV compared to the parental ncp CSFV. The amount of viral RNA is shown in Figure 4B per ng of total cellular RNA to compensate for the effect of lower cell mass in the dying cultures infected with the cp CSFV. In contrast, the amount of viral RNA in the cell culture medium was calculated in GE per ml in Figure 4C to allow direct comparison with the TCID_50_ values of the respective CSFV strains. However, comparable RNA levels were found in ncp CSFV and cp CSFV supernatants suggesting that strong cell lysis in the case of the cp CSFVs resulted in the release of intracellular viral RNA. 

**Figure 5:**
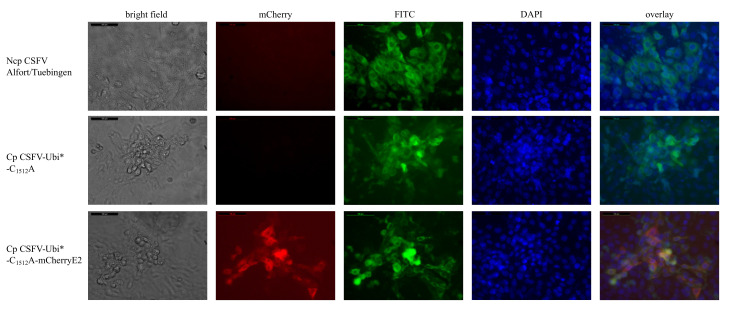
**Focus/plaque size analysis of the different ncp and cp CSFV strains.** SK-6 cells were infected with the respective viruses, as indicated on the left. Cells were stained with the E2-specific Mab A18 and FITC-conjugate. Nuclei were counterstained with DAPI and mCherry, and fluorescence was recorded. Cytopathic effects of the cp CSFVs were documented in bright-field images. The images were combined in overlay for direct comparison. All images were taken at 40× magnification with a superimposed virtual scale bar (100 µm).

The plaque or focus size of cp CSFV-Ubi*-C_1512_A, cp CSFV-Ubi*-C_1512_A-mCherryE2 and ncp CSFV Alfort/Tuebingen was compared to investigate their cell culture spread. SK-6 cells were infected with 10 PFU/FFU of the respective virus. After 2 h of infection, the infection medium was removed and replaced with 1% carboxymethyl cellulose medium to prevent the long-distance spread of viral particles. Focus and plaque size was determined by counting the infected cells within single foci or plaques using immunofluorescence 48 h after infection. In Figure 5, we show representative foci of the ncp and cp virus strains, demonstrating a clear reduction of the plaque size for the cp CSFVs in direct comparison to the parental ncp strain. A statistical analysis of the data is shown in Supplementary Figure S2. Foci of ncp CSFV Alfort/Tuebingen consisted of 128 cells on average (95% CI +/− 7.7), plaques of cp CSFV-Ubi*-C_1512_A consisted of 55 cells (95% CI +/− 2.4) and the plaques of cp CSFV-Ubi*-C_1512_A-mCherryE2 contained only 46 cells (95% CI +/− 2.6).

### 3.5. Characterization of Viral Protein Expression and Polyprotein Processing

**Figure 6:**
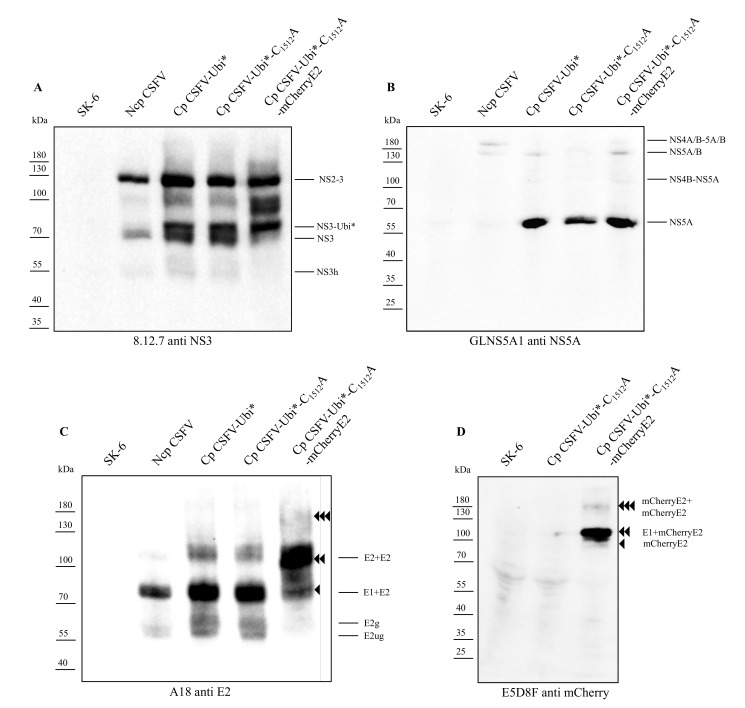
Protein processing profiles. SK-6 cells were harvested 24 h post-transfection together with mock-transfected control cells (SK-6, lane 1). Cell lysates were subjected to SDS-PAGE, blotted onto nitrocellulose membranes, and incubated with mouse monoclonal antibodies as indicated below. (**A**) Proteins detected with Mab 8.12.7 directed against the helicase domain of NS3. An additional band of Ubi*-NS3 is visible in the cp CSFV-Ubi* strains. (**B**) Proteins detected with GLNS5A1 against NS5A. Mature NS5A is only visible in the cp CSFV-Ubi* strains. (**C**) Proteins detected with A18 against E2. An overall stronger expression of viral E2 antigens is apparent in cp CSFV-Ubi* infected cells. The mCherryE2 fusion protein species in cp CSFV-Ubi*-C_1512_A-mCherryE2 show a size increase of 27 kDa as, indicated by arrowheads on the right. (**D**) Proteins detected with E5D8F against mCherry. A broad range protein ladder is presented on the left. Known pestiviral protein species were identified by their apparent molecular weight as indicated on the right side.

We also analyzed the protein expression of the different CSFV strains at 24 h post-transfection. The immunoblot against the helicase domain of NS3 in Figure 6A showed the well-known NS3 species with a prominent band at 125 kDa (NS2-3), a weaker band at 75 kDa (mature NS3) and very weak bands at 100 kDa (NS2*-NS3), as well as 55 kDa (free helicase domain of NS3, NS3h) in the ncp CSFV infected cells. The cp CSFV infected cells present an overall stronger NS protein expression with strong bands at 125 kDa (NS2-3), 82 kDa (Ubi*-NS3) and 75 kDa (mature NS3). Weaker bands were visible at 100 kDa (NS2*-NS3) and 55 kDa (NS3h). An additional band of about 90 kDa was visible in the cp CSFV-Ubi*-C_1512_A-mCherryE2 strain, which has not been characterized so far. The elevated NS protein expression levels and the efficient NS protein processing became apparent by the immunodetection of NS5A in Figure 6B. While solely the weak bands of two precursor molecules of about 175 kDa (NS4A/B-NS5A/B) and 140 kDa (NS5A/B) are visible in the ncp CSFV, an additional very strong band at 57 kDa (mature NS5A) and a weak band at 100 kDa (NS4B-NS5A) were apparent in the cp CSFV strains. The detection of E2 proteins in Figure 6C resulted in a strong band at 80 kDa (mature E1-E2 heterodimer) and weak bands at 57 kDa (unglycosylated E2), 60 kDa (glycosylated E2) and 110 kDa (E2-E2 homodimer) in ncp CSFV. Stronger expression of the same E2 protein species was seen in the cp CSFV strains, while a shift of the bands was visible for the cp CSFV-Ubi*-C_1512_A-mCherryE2 strain with a strong band at 110 kDa (E1-mCherryE2 heterodimer) and weaker bands at 80 kDa (unglycosylated mCherryE2) and 160 kDa (mCherryE2-mCherryE2 homodimer). The unglycosylated mCherryE2 with 80 kDa, a strong band at 110 kDa (E1-mCherryE2 heterodimer) and a weak band with 160 kDa (mCherryE2-mCherryE2 homodimer), were also seen in the immunoblot in Figure 6D using an anti mCherry antibody.

### 3.6. SVNA with a Dual Reporter CSFV as a Valuable Tool for Indirect CSFV Diagnostics

The cp CSFV-Ubi*-C_1512_A-mCherryE2 strain was designed as a novel tool for the indirect detection of CSFV infections using a fluorescence-verified plaque reduction assay. For the validation of this potential application, SVNAs with cp CSFV-Ubi*-C_1512_A-mCherryE2 and ncp CSFV Alfort/Tuebingen were performed in parallel following the standard procedure. Defined positive and negative reference antisera were tested (Table 2). Virus infection in non-protected cell cultures was detectable 48 h post infection in cp CSFV-Ubi*-C_1512_A-mCherryE2 by a strong red fluorescence of defined plaques within the monolayer shown for all cells of the SVNA in Supplementary Figure S3. Evaluation of ncp CSFV and cp CSFV-Ubi*-C_1512_A-mCherryE2 SVNA was compared after immunofluorescence staining using an E2 specific antibody. The evaluation of the cp CSFV-Ubi*-C_1512_A-mCherryE2 SVNA using plaque emergence, the mCherry reporter and E2 specific immunostaining was demonstrated. Furthermore, similar neutralization profiles of this double reporter CSFV and its parental ncp CSFV strain were also shown.

## 4. Discussion

In the field, helper-independent cp pestivirus strains regularly emerge in BVDV infected cattle but have never been isolated from CSFV infected pigs [60]. This circumstance may be explained by the fact that cp strains of BVDV arise from mutations in persistently infected, immunotolerant animals from their ncp progenitor viruses. In contrast, persistent CSFV infections are rather rare in pigs, and most PI piglets die quickly due to the higher virulence of CSFV. However, defective interfering cp subgenomes of CSFV have been isolated in the past from pigs and CSFV infected cell cultures [35]. Previous studies on the molecular basis of the biotype switch uncovered that the cp strains of BVDV produced greater amounts of mature NS3, whereas only the uncleaved NS2-3 was detectable in the parental ncp strains. In this context, the greater amount of mature NS3 resulted in increased processing of the NS3-protease substrate molecules and thus a higher replication rate. No increase in progeny virus production was observed in the cp strains because of the importance of the NS2-3 precursor molecule for particle formation. It was shown that temporal regulation of NS2-3 cleavage is mediated by the cellular Jiv protein, only available in the early phase of infection as a cofactor for the NS2 autoprotease [10].

Reverse genetic systems have been used to create artificial cp strains of CSFV using cp BVDVs as templates. In a first attempt, helper virus-independent cp CSFVs were generated by in vivo RNA recombination. Therefore, synthetic, non-replicative subgenomic RNAs (DIs) were transfected in cells pre-infected with an ncp CSVF. The emerging helpervirus-independent cp CSFVs were isolated by subsequent plaque purification. This approach yielded an 18.1 kb long cp CSFV strain (CP G1), in which a complete DI sequence was integrated at the 3′-end of the ORF. However, CP G1 was genetically unstable and reverted completely during passaging into the parental replicative subgenomic DIs [36].

The first genetically stable cp CSFV was presented in 2008 using genomic integration of a complex Jiv insertion of 1539 nucleotides derived from the cp BVDV strain CP8. The resulting cp CSFV-Jiv was tested in an animal experiment. It showed no reversion to wild-type-like genomes, was completely avirulent and induced a strong protective immune response. The cp CSFV-Jiv was proposed as a suitable vaccine candidate [37]. However, a conceptual disadvantage of a modified live vaccine, which is attenuated by the insertion of Jiv, is that loss of function mutations in the Jiv gene or deletions might occur, which will lead to the emergence of wild-type-like viruses. In this study, we aimed to test a different approach allowing functional separation of NS2-3 and NS3 by insertion of a ubiquitin gene as an auxiliary processing signal together with the duplication of the NS3 to NS4B* sequences. Although many BVDVs have been isolated that exhibit genetically stable duplications, no comparable cp CSFV strains have been generated and presented so far. Large homologous sequences are often not stable in bacterial plasmids hindering the engineering of such stains. These difficulties were circumvented in our study by using a BAC-backbone for the reverse genetics system and by the stabilization of the sequence duplication in the RNA virus genome after rescue by the inactivation of the NS2-autoprotease.

First, we generated a cp CSFV-DI-Ubi* by exchanging the cassette encoding N^pro^ to NS2 protein genes against Ubi*, a ubiquitin fusion protein gene derived from the cp BVDV strain CP Rit. The Ubi* gene has previously been characterized in this well-known temperature-sensitive vaccine strain [18]. The encoded protein is composed of a short fragment from the ribosomal protein S27a (27 aa, ENGKISRLRRECPSDECGAGVFMASHF) upstream of a truncated ubiquitin lacking the first three amino acids (MQI). In this cp BVDV, the Ubi* protein serves as a processing signal releasing Ubi*-NS3, while cellular ubiquitin C-terminal hydrolases (UCHs) cleave the peptide bond between Ubi* and NS3 generating an authentic NS3 N-terminus. The synthetic RNA of the cp CSFV-DI-Ubi* replicated efficiently after transfection, demonstrating the functional activity of the Ubi* on CSFV NS3 maturation as shown in Figure 2. The functional cp CSFV-DI-Ubi* replicase module was inserted in the genome of ncp CSFV upstream of the N-terminal third of NS4B, resembling the genome organization of the BVDV strain CP Rit. Insertion positions of such replicase modules within the N-terminus of NS4B have been found in multiple cp BVDV strains utilizing various processing signals. The cellular mRNA sequences encoding parts of the Golgi-associated ATPase enhancer (GATE-16) were described for the cp BVDV strain CP 721 to provide an example [26,61]. As expected, our cp CSFV-Ubi* replicated efficiently, produced infectious particles and led to cell lysis resembling the phenotype of similar helper virus independent cp BVDVs. The passage of the virus progeny revealed reversion to a wild-type-like genome showing a complete deletion of the duplicated sequences and the Ubi* gene. The genetic instability of cp CSFV-Ubi* most probably resulted from recombination events that occurred between the homologous sequence motives during viral replication. Sequence recombination during virus replication is a frequent event in nature and cannot be completely prevented — even by modifying the duplicated codons as non-homologous recombination might also occur, albeit with a lower frequency. Therefore, we decided to inhibit autonomous replication of possible ncp recombination products by inactivating the NS2 autoprotease. A single amino acid exchange in cp CSFV-Ubi*-C_1512_A, which leads to NS2 autoprotease deficiency, prevented the replication of ncp recombination products and stabilized the cp CSFV. It can be hypothesized that genetically stable cp BVDV strains with duplicated viral sequences known from the field were evolutionarily stabilized in a similar manner. In the selection of the fittest viruses, adaptation processes probably took place compensating the imbalance between mature NS3 and uncleaved NS2-3 by inhibition of the NS2-autoprotease [62]. Cp CSFV-Ubi* has a replicase module independently active from the rest of the genome, and none of the known enzyme functions are important for the packaging function of NS2-3. Hence, cp CSFV-Ubi*-C_1512_A could potentially be further stabilized and optimized by the introduction of additional mutations within NS2 and/or NS3. Since we did not detect reversion in cp CSFV-Ubi*-C_1512_A within 11 passages, we assume that the C_1512_A mutation alone is sufficient to stabilize the cp CSFV-Ubi*. The emergence of mutants carrying larger genomic deletions, which inhibit the growth of helper-independent cp CSFV as DIs, was not observed, probably because the cp CSFV-Ubi*-C_1512_A already replicated its genome with a very high efficacy. The emergence of such DIs was also never observed in cell culture propagation of cp BVDV field strains containing duplicated viral sequences together with insertions of cellular mRNA sequences.

Our new reverse genetics system for a cp CSFV with functionally uncoupled NS2-3 and NS3 proteins allows, for the first time, the study of the independent functions of NS3 and NS2-3 within a single cistron. Future studies will use mutational analyses of separated protease, NTPase and helicase domains as well as of the packaging module to investigate their importance for the different steps in the pestiviral lifecycle. Furthermore, the stabilized cp CSFV showed an enormous antigen expression, which might be beneficial in live attenuated CSFV vaccines. Since this concept can be applied to any CSFV strain, known safe vaccine strains already in use could be modified by inserting stabilized duplications. In this study, we applied the stable cp CSFV-Ubi*-C_1512_A strain to generate a new valuable tool for veterinary CSFV diagnostics. We inserted an additional red fluorescent protein gene (mCherry) at the 5′-end of the E2 ectodomain to produce a dual reporter virus that destroys the cellular monolayer with clearly visible plaques and allows the verification of the virus-related causation of the cell damage by fluorescence microscopy. The application of the cp CSFV-Ubi*-C_1512_A-mCherryE2 in fluorescence verified virus neutralization assays was successfully tested in this study.

Currently, various measures are used to control or eradicate CSFV in different regions of the world. The most important measures in countries with successful CSFV eradication programs are molecular biological diagnostics, which are mainly accomplished by RT(q)-PCRs. Unfortunately, there are still many countries where CSFV is endemic and domestic pig herds must be protected from disease by vaccination. In this regard, SVNA is still a common laboratory system for identifying CSFV affected herds in less developed and economically weak countries. It is also still the “gold-standard” test to confirm questionable reactions in antibody ELISA since a definitive diagnosis can be obtained with the high specificity of the SVNA reaction. It is also possible to distinguish the humoral immune response of pigs against CSFV from reactions against other related viruses, such as BVD or BVDV. The SVNA itself is simple to perform, involves comparatively low equipment and consumable costs, and can be performed even in rural areas in any cell culture laboratory with the necessary safety equipment. In addition, the SVNA can be used to check the protection status of pigs after vaccination, which may be relevant in vaccine development and testing as well as during vaccination campaigns. However, SVNAs cause a very high labor burden when they are performed with ncp viruses, as the cell cultures have to be fixed, permeabilized and immune-assayed before evaluation. The dual reporter CSFV presented in this study can significantly reduce the effort and assay cost. It can also improve the assay performance because the cp CSFV causes a clearly visible cytopathogenic effect, which allows a faster and easier evaluation of SVNA after approximately 48 h as a plaque reduction assay under the light microscope. In case of questionable results, the reactions can be validated by the fluorescence of the infected cells under a fluorescence microscope. Such doubtful reactions occur, for example, when the sera to be tested are toxic to the cell culture system at lower dilution levels, which is frequently observed in sera from diseased animals. In contrast to cp CSFV-Ubi*-C_1512_A-mCherryE2, which causes a strong fluorescence signal only in cells killed by the cp CSFV infection, immunofluorescence staining is often inconclusive in such cases because the dead cells are washed away during the staining steps, and a nonspecific background staining occurs in the remnant cells. LED technology has greatly reduced the purchase price and maintenance costs of fluorescence microscopes [63]. Due to plaque formation and very strong fluorescence reporter activity, the use of cp CSFV-Ubi*-C_1512_A-mCherryE2 could reduce costs and improve the speed and reliability of SVNA against CSFV. The stabilized cp CSFV-Ubi*-C_1512_A still has to be tested in animal experiments to confirm and document its avirulence and immunogenicity, which could be inferred from analogies with cp CSFV-Jiv and various known cp BVDV strains. Given the high cost of such animal studies, the authors of this article cannot perform these tests without industrial collaborators or external funding. Due to the strong antigen expression, the virus may also be suitable as a live vector for the expression of foreign genes in wild boar. In summary, we generated a cytopathogenic CSFV strain rationally stabilized by a point mutation, which has many different potential applications in basic research, prophylaxis, and diagnostics.

## 5. Patents

The author B.L. is the inventor of a patent on pestiviral marker vaccines (WO 2014033149 A1, Marker vaccine).

## Figures and Tables

**Table 1:** Oligonucleotides used in this study.

Name of the Oligonucleotide*	Sequence
Ubi*_forw	5′-GAGAATGGCAAAATCAGTCGCCTTC-3′
Ubi*_rev	5′-CCCACCACGAAGTCTCAACACTAG-3′
CSFV-353-Ubi*-GA_forw	5′-ATCTCTGCTGTACATGGCACATGGAGGTACATGGCACATGGGAGAATG-3′
CSFV-5163-Ubi*-GA_rev	5′-CTTGCAAACAGCTGGCCCCCCACCACGAAGTCTCAAC-3′
CSFV-378_rev	5′-CTCCATGTGCCATGTACAGCAGAGAT-3′
CSFV-5140_forw	5′-GGGCCAGCTGTTTGCAAGAAGG-3′
BAC-SP6_rev	5′-GTATAGTGTCACCTAAATCGTTACAATTCACTGGCCGTCG-3′
CSFV-12271-BAC-GA_forw	5′-GACTAAGGTAATTTCCTAACGGCCCTAAATAGCTTGGCGTAATCATGGTC-3′
SP6_forw	5′-ACGATTTAGGTGACACTATAG-3′
Ubi*-CSFV-7776-GA_rev	5′-GAAGGCGACTGATTTTGCCATTCTCTTGTTGTGTTTCTGTGTCTCCTG-3′
CSFV-12295_rev	5′-GGGCCGTTAGGAAATTACCTTAGTC-3′
CSFV-4891-A1512_forw	5′-GGACCACCAGTGGTCGCCGGTATGACCCTAGCCGATTTC-3′
CSFV-2442_rev	5′-CCGCCCTTGTGCCCCGGTCACCAGCAGCAGCC-3′
CSFV-2443_forw	5′-CTAGCCTGTAAGGAAGACTACAGGTATGCGATC-3′
CSFV-2424-mCherry-GA_forw	5′-GACCGGGGCACAAGGGCGGGTGAGCAAGGGCGAGGAGGATAAC-3′
CSFV-2465-mCherry-GA_rev	5′-CTGTAGTCTTCCTTACAGGCTAGCTTGTACAGCTCGTCCATGCCG-3′
CSFV-RT-qPCR-99_forw (Hoffmann et al. 2005)	5′-ATGCCCAYAGTAGGACTAGCA-3′
CSFV-140-probe_forw (Hoffmann et al. 2005)	FAM-5′-TGGCGAGCTCCCTGGGTGGTCTAAGT-3′-TAMRA
CSFV-RT-qPCR-191_rev (Hoffmann et al. 2005)	5′-CTACTGACGACTGTCCTGTAC-3′
CSFV-5694_rev	5′-GAGCTTGGTTGGTTTGGAATCC-3′
CSFV-7381_forw	5′-GCTCAGGGGGATGTGCAGAGATGTG-3′
CSFV-8421_rev	5′- TAGCTGGCGAATTTTTCCCTCAC-3′
mCherry_forw	5′-ATGGTGAGCAAGGGCGAGGAG-3′
mCherry_rev	5′-CTTGTACAGCTCGTCCATGC-3′

Nucleotide numbers in the oligonucleotide names refer to the nucleotide number in CSFV strain Alfort/Tuebingen (GenBank J04358.2). The abbreviations “forw and rev” mean forward and reverse, referring to the viral plus-strand.

**Table 2:** Results of comparative SVNAs using ncp CSFV and a fluorescent cp CSFV.

Serum ID	SND_50_/mL Ncp CSFV Alfort-Tübingen	SND_50_/mL Cp CSFV-Ubi*-C_1512_A-mCherryE2
2016/01/0441/027	1/370	1/370
2008/03/0225/032	1/370	1/370
2006/07/0056/086	1/20,500	1/20,500
Neg. control serum	n.d. *	n.d. *

* n.d. means “not detected”—no virus neutralization was observed.

## Data Availability

All data analyzed or generated during this study are included in the manuscript.

## Supplementary Materials

The following are available online at https://www.mdpi.com/article/10.3390/v13071209/s1, Figure S1: Sequence of the NS4B gene from cp CSFV-Ubi* after passage 5, Figure S2: Focus and plaque size of the different CSFV strains, Figure S3: Documentation of the fluorescence-verified plaque reduction assay.
