# Supplementary material for: Characterization of a Cytopathogenic Reporter CSFV
Source: Viruses. 2021 Jun 23;13(7):1209. doi: 10.3390/v13071209 (PMC8310069; doi:10.3390/v13071209)

Figure S1

CSFV-7381_for

5‘-**GCTCAGGGGGATGTGCAGAGATGTG**TGGAAGCAGTGACCAATTATGCGAGAGAGGGCATCCAATTCATGAAGTCGCAGGCACTGAA
AGTGAGAGAAACCCCTACCTATAAAGAGACAATGAACACCGTGGCAGATTATGTGAAAAAGTTTATTGAGGCACTGACGGATAGCAAGG
AAGACATCATTAAATATGGGCTGTGGGGGCACATACGGCATTGTATAAGAGCATTGGTGCCAGGCTTGGTCACGAAACCGCGTTCGCA
ACTCTAGTTGTGAAGTGGTTGGCATTTGGGGGGAGTCAATATCAGACCACATAAAGCAAGCGGCCACAGACTTGGTCGTTTATTACAT
TATTAACAGACCTCAATTCCCAGGAGACACAGAAACACAACAAGAAGGGAGAAAATTTGTTGCCAGCCTGCTAGTCTCAGCTCTAGCGA
CTTATACATACAAGAGCTGGAACTACAATAATCTGTCCAAAATAGTTGAACCGGCTTTGGCTACCCTGCCCTATGCCGCTAAAGCCCTC
AAGCTATTTGCTCCTACCCGACTGGAGAGCGTTGTCATACTGAGCACTGCAATCTACAAAACATACCTATCAATAAGGCGAGGCAAAAG
TGATGGTCTGCTAGGTACAGGGGTTAGCGCGGCCATGGAAATTATGTCACAAAACCCAGTATCTGTGGGTATAGCAGTTATGCTAGGGG
TAGGGGCTGTAGCAGCCCACAATGCAATTGAAGCCAGTGAGCAAAAAAGAACACTACTTATGAAAGTCTTTGTGAAAAACTTCTTAGAC
CAGGCCGCCACCGACGAACTAGTCAAAGAGAGCCCTGAGAAAATAATAATGGCTTTGTTCGAAGCGGTGCAAACGGTGGGCAACCCTCT
TAGATTAGTGTACCACCTCTATGGAGTTTTCTATAAAGGGTGGAAGCAAAAGAGTTGGCCCAAAGAACAGCCGGCAGGAACCTTTTCA
CCTTGATAATGTTCGAGGCTGTGGAACTACTGGGAGTAGACA**GTGAGGGAAAAATTCGCCAGCTA**-3‘

CSFV-8421_rev

Figure S1: Sequence of the NS4B gene from cp CSFV-Ubi* after passage 5. After virus infection, the surviving cells were trypsinized and total RNA extracted. An RT-PCR amplicon was generated using oligonucleotides CSFV-7381_for and CSFV-8421 rev. The PCR product was inserted into a T-vector and sequenced using M13 and M13 rev. A pinpoint reversion by homologous recombination to the parental ncp CSFV strain was documented.

Figure S2

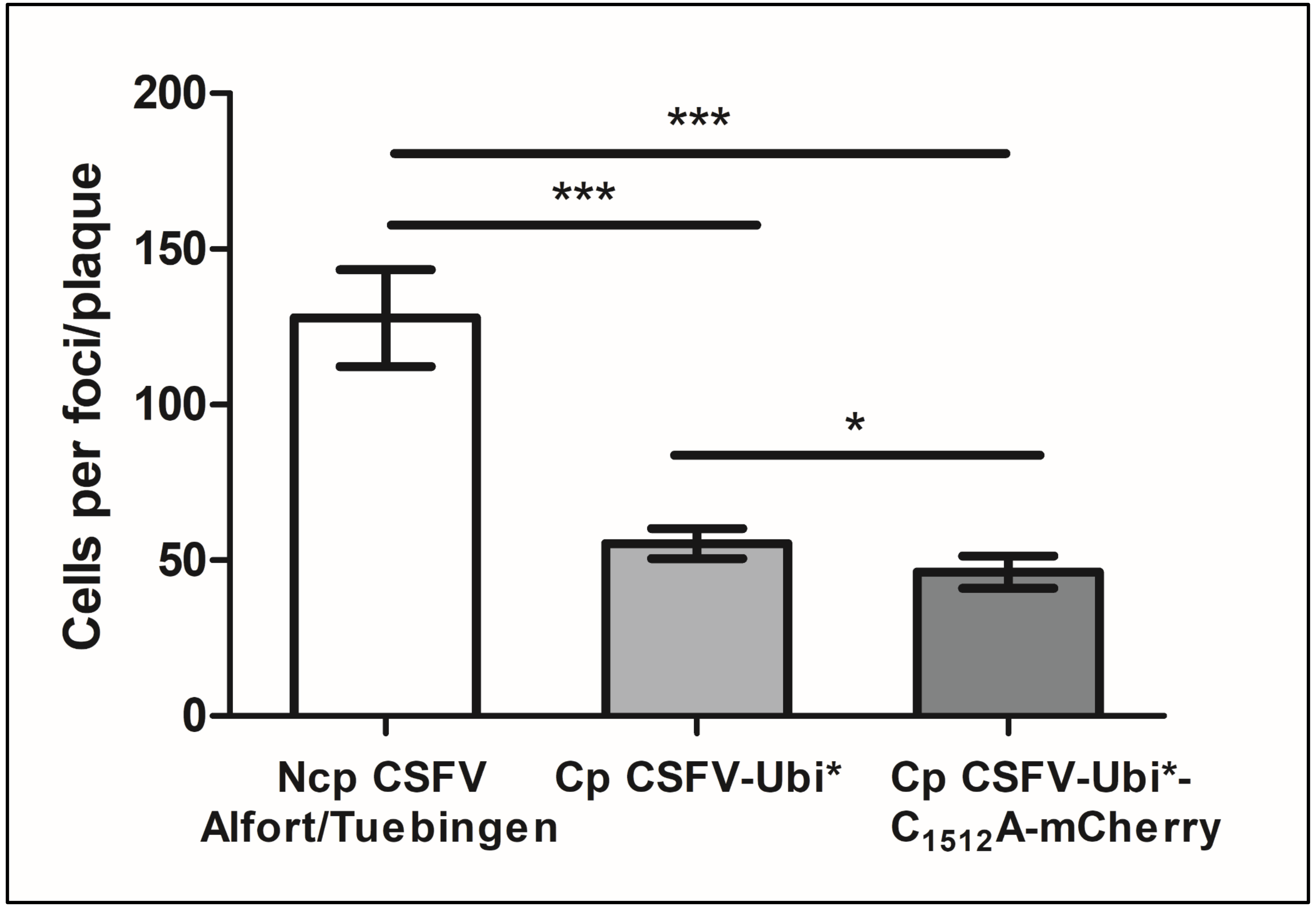

Figure S2: Focus and plaque size of the different CSFV strains. After infection of SK-6 cells, the supernatant was removed and replaced by fresh DMEM containing 1% car-boxymethyl cellulose. The infected cells of 50 individual foci were counted for each virus strain, the mean focus size was calculated, and the 95% confidence interval was determined. The values were compared in the unpaired T-test.

A SVNA for positive serum 2016/01/0441/027

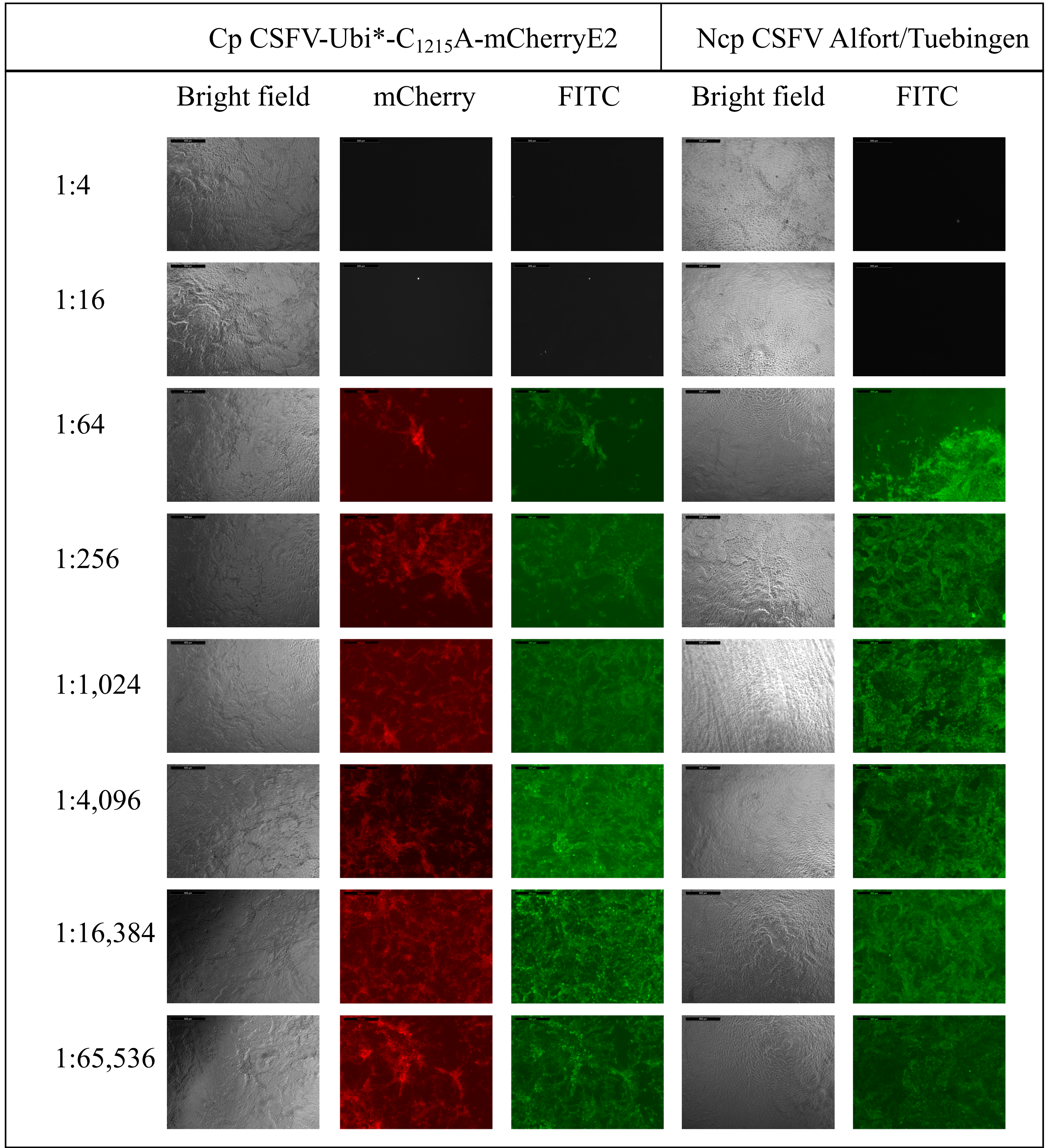

Figure S3: Documentation of the fluorescence-verified plaque reduction assay. (**A**) Low positive serum 2016/01/0441/027. (**B**) Low positive serum 2008/03/0225/032. (**C**) High positive serum 2006/07/0056/086. (**D**) Negative control serum. The sera were tested against the viruses cp CSFV-Ubi*-C1512A-mCherryE2 (left) and ncp CSFV Alfort/Tuebingen (right). Indicator cells were fixed 48 h post infection and stained with A18 anti E2 and FITC conjugate. A representative area of each 96-well was documented in brightfield, red and green fluorescence channels at 4x magnification. A scale bar (500 µm) is used for comparison between images.

B SVNA for positive serum 2008/03/0225/032

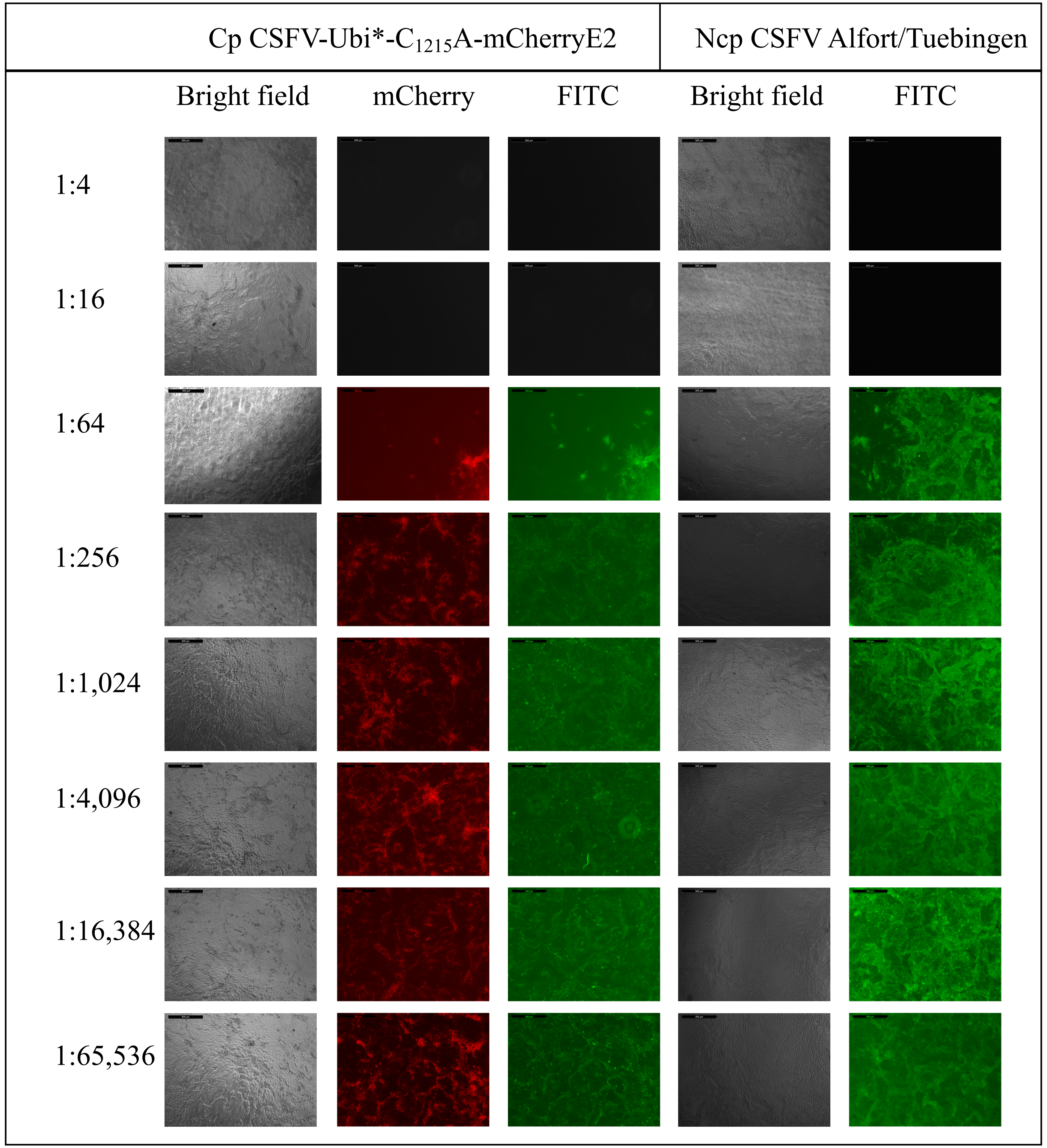

C SVNA for positive serum 2006/07/0056/086

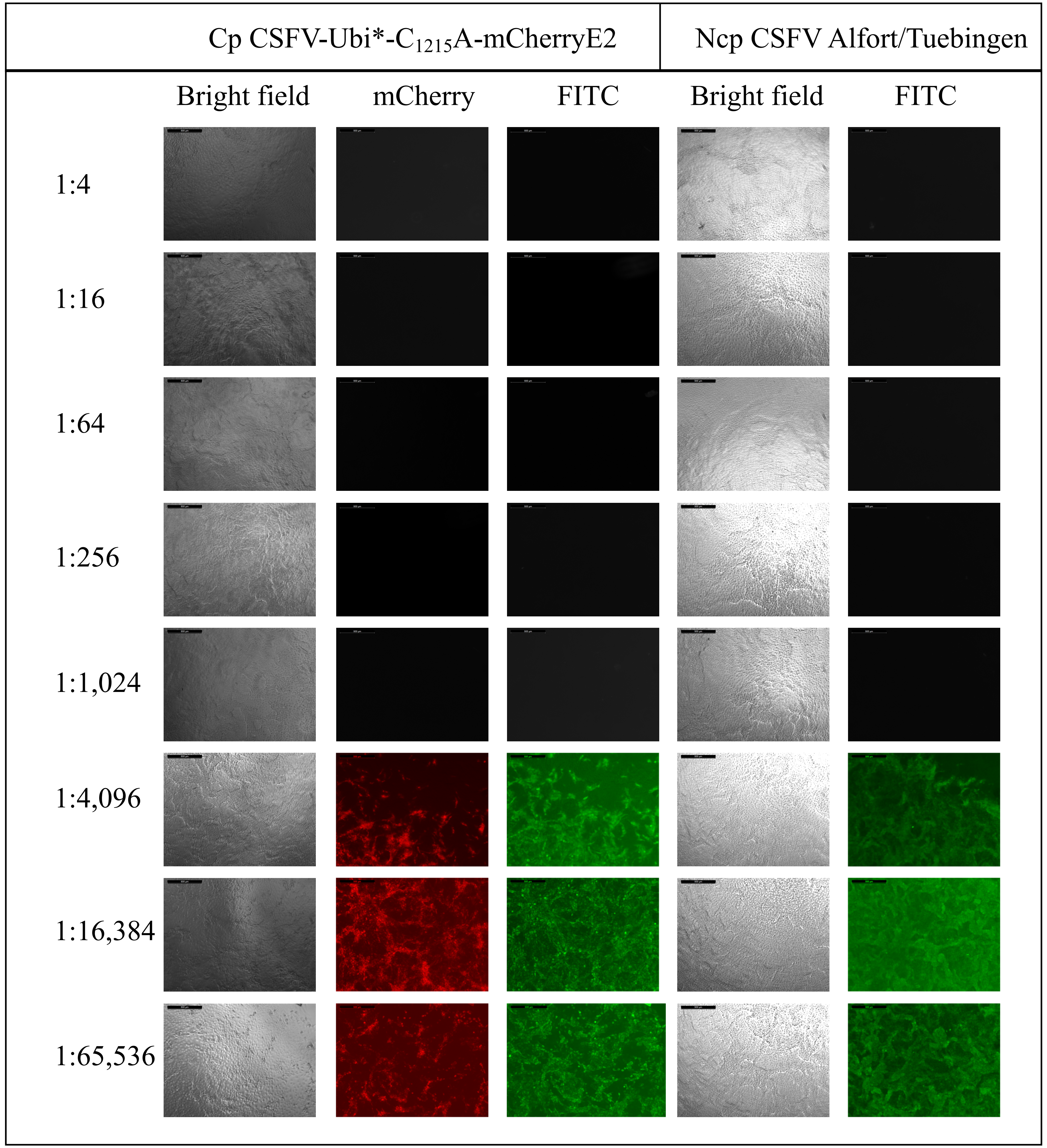

D SVNA for the negative control serum

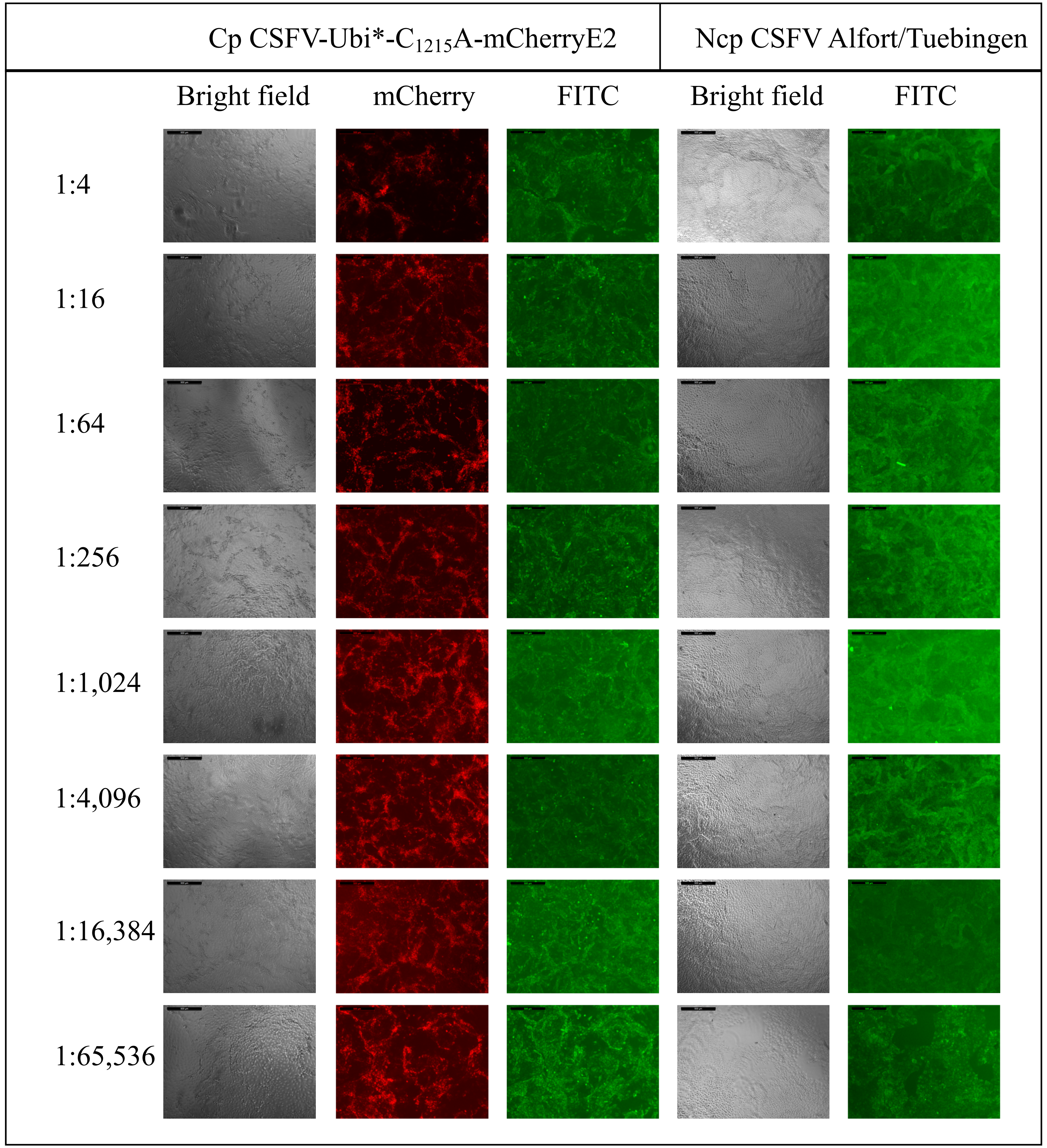

Supplement: Supplementary file 1 [file viruses-13-01209-s001.zip › viruses-1246100-supplementary.pdf]
